# Supplementary figures and images for: GenomePeek—an online tool for prokaryotic genome and metagenome analysis
Source: PeerJ. 2015 Jun 16;3:e1025. doi: 10.7717/peerj.1025 (PMC4476108; doi:10.7717/peerj.1025)

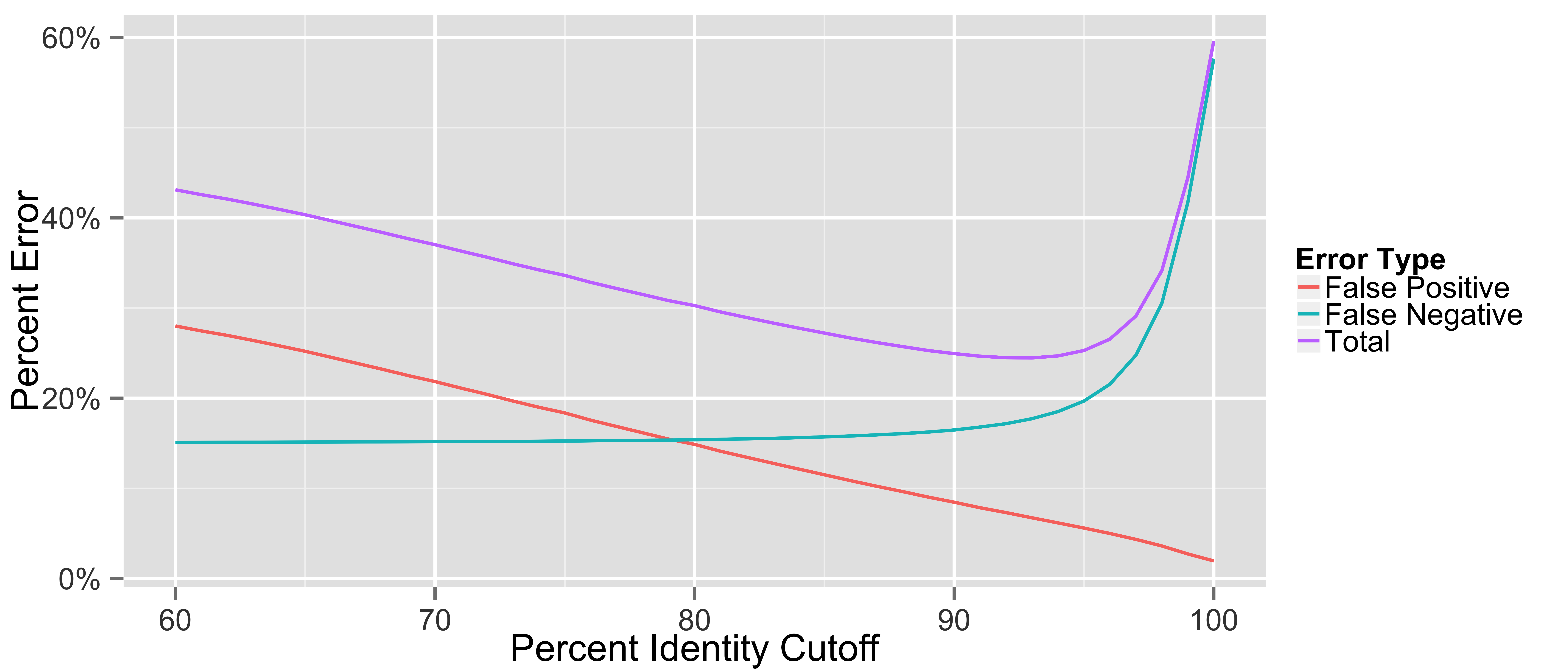

Supplement: Figure S1 — Varying the percent identity cutoff on MG-RAST and its effect on the error rate when identifying the species of the FAMES metagenomic reads. [file peerj-03-1025-s001.png]

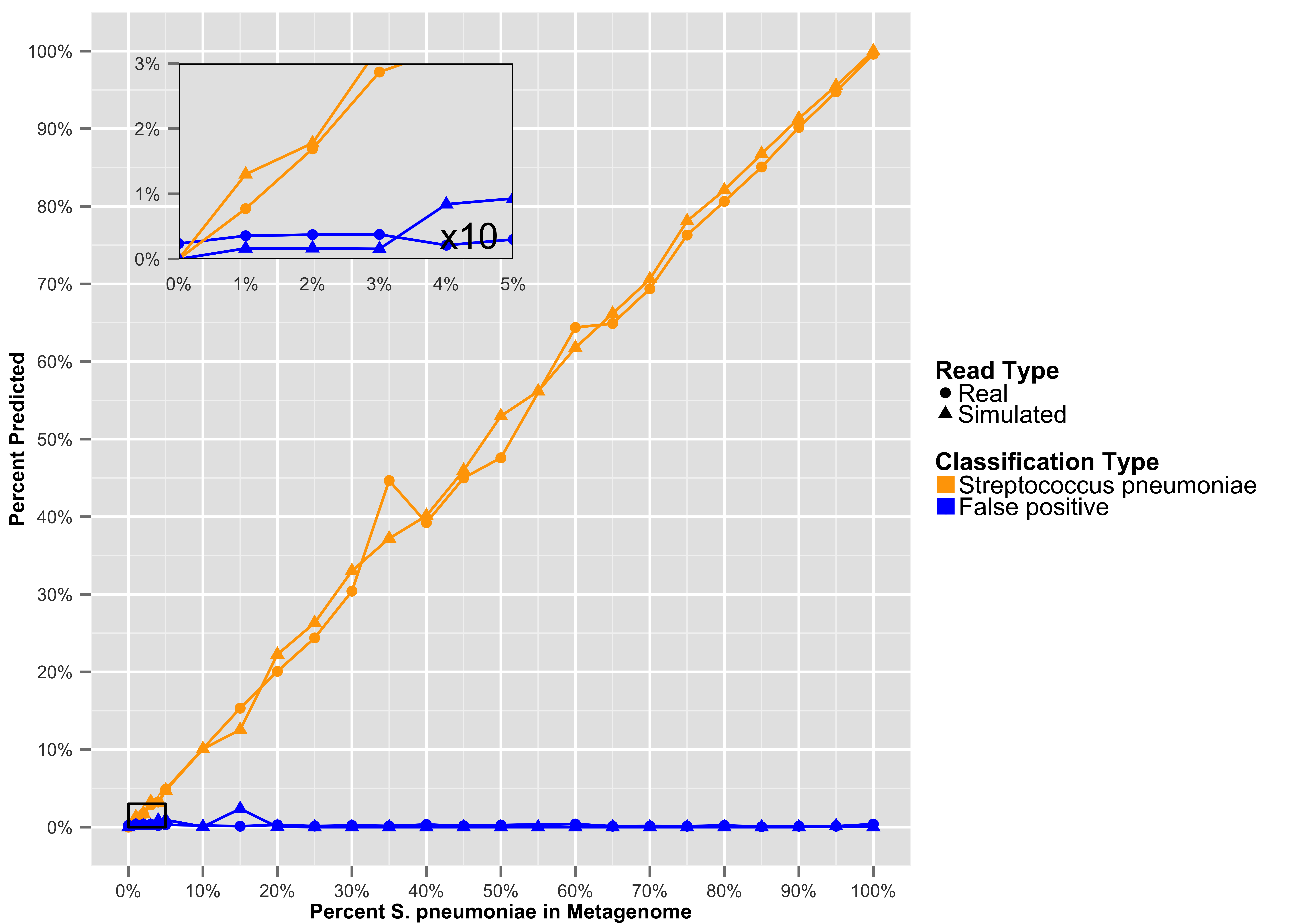

Supplement: Figure S2 — Summed false positive error rates when GenomePeek is used to analyze sequencing files of S. pyogenes that have been contaminated with low levels of S. pneumoniae, and using only the genes, RecA, RpoB, and groEL. [file peerj-03-1025-s002.png]
