# Supplementary material for: GenomePeek—an online tool for prokaryotic genome and metagenome analysis
Source: PeerJ. 2015 Jun 16;3:e1025. doi: 10.7717/peerj.1025 (PMC4476108; doi:10.7717/peerj.1025)
Supplement: Table S1 [file peerj-03-1025-s003.docx]

Supplementary Table 1: False positive rates when identifying the species of artificial sequencing data and real sequencing data.

|  | **GenomePeek**  **(%)** | **MG-RAST**  **(%)** | **MetaPhlAn**  **(%)** | **MEGABLAST**  **(%)** |
| --- | --- | --- | --- | --- |
| **C. jejuni** |  |  |  |  |
| Artificial Data | 0.00 | 2.73 | 0.00 | 2.30 |
| Real Data | 0.04 | 2.75 | 0.00 | 2.59 |
| **C. difficile** |  |  |  |  |
| Artificial Reads | 0.01 | 0.45 | 0.00 | 0.75 |
| Real Data | 0.03 | 5.28 | 0.00 | 7.17 |
| **E. coli** |  |  |  |  |
| Artificial Reads | 0.00 | 58.68 | 0.00 | 3.60 |
| Real Data | 0.54 | 58.88 | 0.01 | 3.07 |
| **M. tuberculosis** |  |  |  |  |
| Artificial Reads | 10.25 | 47.20 | 0.00 | 10.18 |
| Real Data | 15.46 | 48.40 | 0.02 | 13.25 |
| **N. meningitidis** |  |  |  |  |
| Artificial Reads | 0.00 | 21.50 | 0.00 | 2.88 |
| Real Data | 0.18 | 19.17 | 0.00 | 1.91 |
| **S. enterica** |  |  |  |  |
| Artificial Reads | 0.00 | 32.28 | 0.00 | 0.83 |
| Real Data | 0.38 | 31.93 | 0.00 | 1.64 |
| **S. aureus** |  |  |  |  |
| Artificial Reads | 0.00 | 28.94 | 0.00 | 1.23 |
| Real Data | 0.02 | 29.06 | 0.00 | 1.15 |
| **S. pneumoniae** |  |  |  |  |
| Artificial Reads | 0.00 | 11.28 | 0.01 | 1.66 |
| Real Data | 0.15 | 10.33 | 0.01 | 1.45 |
| **S. pyogenes** |  |  |  |  |
| Artificial Reads | 0.00 | 9.35 | 0.00 | 4.90 |
| Real Data | 0.15 | 9.17 | 0.00 | 4.42 |
| **V. cholerae** |  |  |  |  |
| Artificial Reads | 0.02 | 11.35 | 0.00 | 9.64 |
| Real Data | 0.07 | 13.39 | 0.00 | 9.78 |
