# Supplementary material for: GenomePeek—an online tool for prokaryotic genome and metagenome analysis
Source: PeerJ. 2015 Jun 16;3:e1025. doi: 10.7717/peerj.1025 (PMC4476108; doi:10.7717/peerj.1025)
Supplement: Table S2 [file peerj-03-1025-s004.docx]

Supplementary Table 2: The predicted abundances of S. pyogenes and S. pneumoniae in artificial contaminated sequence data.

|  | **Simulated Reads** | | **Real Reads** | |
| --- | --- | --- | --- | --- |
| **Abundance of S. pneumoniae in metagenome**  **(%)** | **Predicted Abundance of S. pneumoniae**  **(%)** | **False Positive Abundance**  **(%)** | **Predicted Abundance of S. pneumoniae**  **(%)** | **False Positive Abundance**  **(%)** |
| 0 | 0.00 | 0.00 | 0.00 | 0.20 |
| 1 | 0.39 | 0.07 | 0.53 | 0.35 |
| 2 | 0.55 | 0.09 | 1.17 | 0.45 |
| 3 | 0.95 | 0.12 | 1.89 | 0.60 |
| 4 | 1.02 | 0.34 | 2.19 | 0.33 |
| 5 | 1.42 | 0.36 | 3.23 | 0.40 |
| 10 | 3.38 | 0.37 | 6.24 | 0.93 |
| 15 | 4.40 | 1.24 | 9.93 | 0.55 |
| 20 | 8.09 | 0.86 | 14.10 | 1.75 |
| 25 | 12.69 | 0.21 | 16.14 | 1.57 |
| 30 | 12.24 | 1.24 | 18.87 | 2.12 |
| 35 | 18.31 | 0.41 | 29.63 | 2.98 |
| 40 | 20.63 | 0.66 | 50.35 | 0.83 |
| 45 | 31.79 | 0.66 | 29.70 | 3.93 |
| 50 | 34.04 | 3.76 | 54.06 | 2.65 |
| 55 | 40.80 | 1.34 | 73.99 | 4.25 |
| 60 | 84.95 | 0.36 | 39.90 | 3.02 |
| 65 | 86.33 | 0.34 | 41.10 | 4.96 |
| 70 | 85.46 | 0.14 | 88.95 | 0.10 |
| 75 | 92.82 | 0.26 | 45.59 | 5.92 |
| 80 | 94.26 | 0.04 | 87.02 | 3.71 |
| 85 | 95.78 | 0.08 | 95.01 | 0.05 |
| 90 | 97.39 | 0.04 | 96.82 | 0.07 |
| 95 | 98.65 | 0.08 | 98.36 | 0.06 |
| 100 | 100.00 | 0.00 | 99.88 | 0.12 |
