# Supplementary material for: GenomePeek—an online tool for prokaryotic genome and metagenome analysis
Source: PeerJ. 2015 Jun 16;3:e1025. doi: 10.7717/peerj.1025 (PMC4476108; doi:10.7717/peerj.1025)
Supplement: Table S3 [file peerj-03-1025-s005.docx]

Supplementary Table 3: Error rates of classifying the genus of reads of a metagenomes, by various tools.

|  | **GenomePeek** | **MG-RAST** | **MetaPhlAn** | **MEGABLAST** |
| --- | --- | --- | --- | --- |
| **simLC** |  |  |  |  |
| False Positive (%) | 1.98 | 0.50 | 0.00% | 0.70 |
| False Negative (%) | 6.81 | 0.00 | 63.46% | 0.00 |
| **simMC** |  |  |  |  |
| False Positive (%) | 2.30 | 0.70 | 0.00% | 0.60 |
| False Negative (%) | 5.40 | 0.00 | 75.87% | 0.00 |
| **simHC** |  |  |  |  |
| False Positive (%) | 0.72 | 0.70 | 0.00% | 1.20 |
| False Negative (%) | 3.86 | 0.00 | 85.46% | 0.00 |
| **HMP Illumina Even** |  |  |  |  |
| False Positive (%) | 9.17 | 2.94 | 0.00% | 1.78 |
| False Negative (%) | NA* | NA* | NA* | NA* |
| **HMP Illumina Staggered** |  |  |  |  |
| False Positive (%) | 11.10 | 13.78 | 0.02% | 2.06 |
| False Negative (%) | NA* | NA* | NA* | NA* |
| **HMP 454 Even** |  |  |  |  |
| False Positive (%) | 6.33 | 2.79 | 0.00% | 1.37 |
| False Negative (%) | NA* | NA* | NA* | NA* |
| **HMP 454 Staggered** |  |  |  |  |
| False Positive (%) | 7.49 | 1.63 | 0.00% | 1.40 |
| False Negative (%) | NA* | NA* | NA* | NA* |
| **9MM-A** |  |  |  |  |
| False Positive (%) | 0.48 | 18.00 | 0.00% | 5.87 |
| False Negative (%) | NA* | NA* | NA* | NA* |
| **9MM-B** |  |  |  |  |
| False Positive (%) | 5.47 | 19.80 | 0.02% | 6.72 |
| False Negative (%) | NA* | NA* | NA* | NA* |

* Not able to calculate a false negative error due to the fact that the actual abundance distribution is not known.
