# Supplementary material for: GenomePeek—an online tool for prokaryotic genome and metagenome analysis
Source: PeerJ. 2015 Jun 16;3:e1025. doi: 10.7717/peerj.1025 (PMC4476108; doi:10.7717/peerj.1025)
Supplement: Table S4 [file peerj-03-1025-s006.docx]

Supplementary Table 4: Error rates of classifying the species of reads of a metagenomes, by various tools.

|  | **GenomePeek** | **MG-RAST** | **MetaPhlAn** | **MEGABLAST** |
| --- | --- | --- | --- | --- |
| **simLC** |  |  |  |  |
| False Positive (%) | 3.54% | 17.20% | 0.00% | 1.40% |
| False Negative (%) | 10.02% | 0.60% | 67.53% | 0.00% |
| **simMC** |  |  |  |  |
| False Positive (%) | 7.42% | 22.90% | 0.00% | 1.20% |
| False Negative (%) | 7.86% | 0.50% | 76.89% | 0.00% |
| **simHC** |  |  |  |  |
| False Positive (%) | 9.74% | 16.10% | 0.00% | 2.30% |
| False Negative (%) | 7.23% | 0.90% | 93.79% | 0.00% |
| **HMP Illumina Even** |  |  |  |  |
| False Positive (%) | 9.17% | 17.80% | 0.36% | 2.57% |
| False Negative (%) | NA* | NA* | NA* | NA* |
| **HMP Illumina Staggered** |  |  |  |  |
| False Positive (%) | 11.10% | 18.94% | 0.24% | 2.85% |
| False Negative (%) | NA* | NA* | NA* | NA* |
| **HMP 454 Even** |  |  |  |  |
| False Positive (%) | 6.33% | 12.50% | 0.57% | 1.69% |
| False Negative (%) | NA* | NA* | NA* | NA* |
| **HMP 454 Staggered** |  |  |  |  |
| False Positive (%) | 7.49% | 24.40% | 1.5% | 2.08% |
| False Negative (%) | NA* | NA* | NA* | NA* |
| **9MM-A** |  |  |  |  |
| False Positive (%) | 0.48% | 25.40% | 0.00% | 6.38% |
| False Negative (%) | NA* | NA* | NA* | NA* |
| **9MM-B** |  |  |  |  |
| False Positive (%) | 5.47% | 37.50% | 0.02% | 8.80% |
| False Negative (%) | NA* | NA* | NA* | NA* |

* Not able to calculate a false negative error due to the fact that the actual abundance distribution is not known.
