# Supplementary material for: GenomePeek—an online tool for prokaryotic genome and metagenome analysis
Source: PeerJ. 2015 Jun 16;3:e1025. doi: 10.7717/peerj.1025 (PMC4476108; doi:10.7717/peerj.1025)
Supplement: Table S5 [file peerj-03-1025-s007.docx]

Supplementary Table 5: Runtimes for the various sequence files across the four different applications used.

|  | **GenomePeek**  **(min)** | **MG-RAST**  **(min)** | **MetaPhlAn**  **(min)** | **MEGABLAST**  **(min)** |
| --- | --- | --- | --- | --- |
| **Simulated Data** |  |  |  |  |
| C. jejuni | 4 | 6028 | 1 | 235 |
| C. difficile | 3 | 6054 | 1 | 258 |
| E. coli | 4 | 6165 | 1 | 318 |
| M. tuberculosis | 4 | 6102 | 1 | 195 |
| N. meningitidis | 4 | 6041 | 1 | 357 |
| S. enterica | 5 | 6145 | 1 | 329 |
| S. aureus | 4 | 6066 | 1 | 288 |
| S. pneumoniae | 4 | 6051 | 1 | 406 |
| S. pyogenes | 4 | 6028 | 1 | 336 |
| V. cholerae | 4 | 6110 | 1 | 339 |
| **Real Data** |  |  |  |  |
| C. jejuni | 460 | 13902 | 1 | 9671 |
| C. difficile | 470 | 14518 | 1 | 10079 |
| E. coli | 33 | 8322 | 1 | 1514 |
| M. tuberculosis | 55 | 14560 | 1 | 979 |
| N. meningitidis | 28 | 5988 | 1 | 1023 |
| S. enterica | 43 | 14584 | 2 | 2903 |
| S. aureus | 28 | 13704 | 3 | 3248 |
| S. pneumoniae | 27 | 6056 | 1 | 1374 |
| S. pyogenes | 94 | 13680 | 1 | 5214 |
| V. cholerae | 24 | 14529 | 1 | 1989 |
| **Metagenomes** |  |  |  |  |
| simLC | 15 | 3485 | 1 | 55 |
| simMC | 16 | 3981 | 1 | 74 |
| simHC | 16 | 4114 | 1 | 79 |
| HMP Illumina Even | 247 | 4371 | 1 | 1510 |
| HMP Illumina Staggered | 274 | 4544 | 1 | 2102 |
| HMP 454 Even | 81 | 38845 | 1 | 794 |
| HMP 454 Staggered | 43 | 38807 | 1 | 993 |
| 9MM-A | 31 | 36180 | 1 | 2111 |
| 9MM-B | 28 | 34800 | 1 | 1488 |
