# Supplementary material for: GenomePeek—an online tool for prokaryotic genome and metagenome analysis
Source: PeerJ. 2015 Jun 16;3:e1025. doi: 10.7717/peerj.1025 (PMC4476108; doi:10.7717/peerj.1025)
Supplement: Table S6 [file peerj-03-1025-s008.docx]

Supplementary Table 6: Metadata for the various sequence files.

|  | **Number of Reads** | **Average Read Length**  **(bp)** |
| --- | --- | --- |
| **Simulated Data** |  |  |
| C. jejuni | 1000000 | 101 |
| C. difficile | 1000000 | 101 |
| E. coli | 1000000 | 101 |
| M. tuberculosis | 1000000 | 101 |
| N. meningitidis | 1000000 | 101 |
| S. enterica | 1000000 | 101 |
| S. aureus | 1000000 | 101 |
| S. pneumoniae | 1000000 | 101 |
| S. pyogenes | 1000000 | 101 |
| V. cholerae | 1000000 | 101 |
| **Real Data** |  |  |
| C. jejuni | 29632084 | 101 |
| C. difficile | 11455106 | 101 |
| E. coli | 3360300 | 231 |
| M. tuberculosis | 7279254 | 101 |
| N. meningitidis | 2098280 | 145 |
| S. enterica | 9597564 | 100 |
| S. aureus | 12499286 | 100 |
| S. pneumoniae | 2962212 | 142 |
| S. pyogenes | 186627336 | 100 |
| V. cholerae | 5585772 | 105 |
| **Metagenomes** |  |  |
| simLC | 97495 | 952 |
| simMC | 114457 | 969 |
| simHC | 116771 | 950 |
| HMP Illumina Even | 6562065 | 75 |
| HMP Illumina Staggered | 7932819 | 75 |
| HMP 454 Even | 1386198 | 397 |
| HMP 454 Staggered | 1225169 | 416 |
| 9MM-A | 7534806 | 76 |
| 9MM-B | 5809246 | 76 |
